# Immobilization of Acetylcholinesterase on Screen-Printed Electrodes. Application to the Determination of Arsenic(III)

**DOI:** 10.3390/s100302119

**Published:** 2010-03-16

**Authors:** Silvia Sanllorente-Méndez, Olga Domínguez-Renedo, M. Julia Arcos-Martínez

**Affiliations:** Departamento de Química, Área de Química Analítica, Facultad de Ciencias, Universidad de Burgos, Plaza Misael Bañuelos s/n, 09001, Burgos, Spain; E-Mails: olgado@ubu.es (O.D.-R.); jarcos@ubu.es (M.J.A.-M.)

**Keywords:** arsenic determination, screen-printed electrode, acetylcholinesterase, biosensor

## Abstract

Enzymatic amperometric procedures for measuring arsenic, based on the inhibitive action of this metal on acetylcholinesterase enzyme activity, have been developed. Screen-printed carbon electrodes (SPCEs) were used with acetylcholinesterase covalently bonded directly to its surface. The amperometric response of acetylcholinesterase was affected by the presence of arsenic ions, which caused a decrease in the current intensity. The experimental optimum working conditions of pH, substrate concentration and potential applied, were established. Under these conditions, repeatability and reproducibility of biosensors were determined, reaching values below 4% in terms of relative standard deviation. The detection limit obtained for arsenic was 1.1 × 10^−8^ M for Ach/SPCE biosensor. Analysis of the possible effect of the presence of foreign ions in the solution was performed. The method was applied to determine levels of arsenic in spiked tap water samples.

## 1. Introduction

Nowadays, environmental pollution caused by metals in different quantities is common, and their traces may often originate from natural as well as anthropogenic sources. Many waters contain high concentration of toxic metals such as arsenic, and excessive concentrations are known to naturally occur in some areas. Natural arsenic contamination is a cause of concern in many countries of the world including Argentina, Bangladesh, Chile, China, India, Mexico, Thailand and the United States of America. The World Health Organization’s (WHO’s) [1] Guideline Value for arsenic in drinking water is 10 μg L^−1^. This figure is limited by the ability to analyze low concentrations of arsenic in water. Many detection methods have been developed for determination of such levels of arsenic. These include atomic fluorescence spectrometry (AFS) [2], atomic absorption spectrometry (AAS) [3], inductively coupled plasma optical emission spectrometry/mass spectrometry (ICP-OES/MS) [4,5] and high-performance liquid chromatography-inductively coupled plasma mass spectrometry (HPLC-ICPMS) [6]. However, most of these techniques are only suitable for laboratory conditions, and additionally, are time-consuming. In fact, these techniques are impractical for on-site screening or for quantification as part of a decision tool owing to their size and high labour and analytical costs. Hence, there is a need for portable analytical systems, which can be met by using electrochemical methods [7]. Electroanalytical techniques bring with them important advantages, such as high sensitivity, low detection limits, relative simplicity, low costs and portable field-based equipment able to determine trace elements. For this reason, electrochemical techniques offer an interesting alternative to methods that are currently in use. Voltammetric methods are among the electrochemical techniques described for the analysis of arsenic. These are relatively widespread, and due to their accuracy and sensitivity, have contributed greatly to its determination at trace level [8,9].

It is well-known that some metals act as enzyme inhibitors. This phenomenon, when it is used to determine these hazardous toxic elements, offers several advantages, among which are sensitivity and specificity. Numerous enzyme inhibition based amperometric sensors have recently appeared in scientific literature for the determination of different metals [10–15]. Acetylcholinesterase, a biological catalyst of primary importance in the transmission of the nerve impulse, is a frequently enzyme used for this purpose [10,16,17].

The possibilities for the amperometric biosensors can be increased by means of replacing the classical electrodes by disposable screen-printed electrodes (SPEs). SPEs present important advantages, such as the elimination of memory effects in the analysis at trace levels, and they appear to be particularly attractive for *in situ* determinations. The construction of SPEs involves the printing of different inks on planar ceramic or plastic supports. The great flexibility of SPEs resides in their high number of possible modifications. In fact, the composition of the inks used in the printing process can be modified by adding substances of a very different nature, such as metals, enzymes, polymers, complexing agents, *etc*.

In the present work, acetylcholinesterase (Ach) based amperometric biosensors were utilized for determination of arsenic(III) based on the inhibition of Ach enzyme activity caused by this metal. To the best of our knowledge, this is the first time that a disposable Ach amperometric biosensor has been used for the high sensitive and selective determination of arsenic. The enzyme was immobilized by covalent linkage on the surface of screen-printed carbon electrodes (SPCEs).

## 2. Results and Discussion

In order to build the Ach/SPE biosensor, several experiments were done with the aim to find the optimum conditions for enzyme immobilization. The maximum inhibition response recorded was reached using the immobilization procedure described [18,19].

The Ach/SPE biosensor produces an amperometric signal, which is sensitive to the concentration of acetylthiocholine iodide. The principle of the determinations is based on the inhibition effect of AsO_3_^3−^ on the activity of the enzyme Ach, immobilized on a SPCEs.

$$\text{Acetylthiocholine iodide}+H_{2}O\overset{\text{Ach}}{\to }\text{thiocholine iodide}+\text{acetic acid}$$

It has been proved that thiocholine, the product of this process, is electroactive. This specie suffers an anodic oxidation, providing a suitable signal for the arsenic determination

**Figure:**
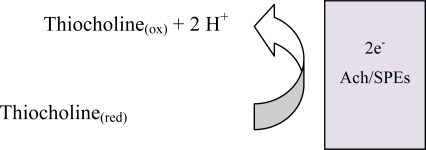


The As(III), interacting with the Ach, inactivates this enzyme; the quantity of thiocholine generated diminishes and the value of the registered oxidation current also decreases as a function of AsO_3_^3−^ concentration, under similar conditions.

As it is well known, arsenate(V) is not an Ach inhibitor, contrary to arsenite(III) [20].

**Figure 1.**
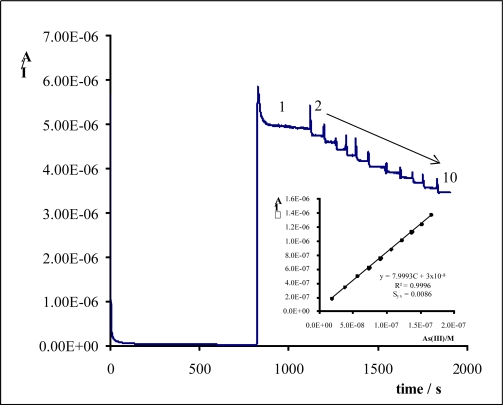
Typical amperometric recording for an acetylthiocholine iodide concentration: (1) 3.64 × 10^−4^ M and consecutive addition of aliquots of As(III) solution into the cell to give an overall concentration of: (2) 1.90 × 10^−8^ M, (3) 5.60 × 10^−8^ M, (4) 7.40 × 10^−8^ M, (5) 9.0 × 10^−8^ M, (6) 1.07 × 10^−7^ M, (7) 1.22 × 10^−7^ M, (8) 1.37 × 10^−7^ M, (9) 1.52 × 10^−7^ M and (10) 1.66 × 10^−7^ M. *E*_ap_ = 0.6 V *vs.* Ag/AgCl, Britton-Robinson buffer pH = 7. The inset shows the relative calibration plot.

As(III) inhibition action was quantitatively evaluated by determining the difference between the steady-state current in the absence of arsenic (I_0_) and the steady-state current in the presence of arsenic (I) (Figure 1). The parameter ΔI (I_0_ − I) depends on acetylthiocholine iodide concentration, applied potential (E_ap_) and pH of the buffer solution. Therefore, it is necessary to optimize all of these variables in order to ensure the quality of the results. Several experiments were carried out at different values of the experimental variables. From these experiments, the following optimum values can be set: concentration of the substrate 3.6 × 10^−4^ M, Britton-Robinson buffer pH 7 and applied potential +0.6 V (*versus* Ag/AgCl), because under these conditions high analytical quality signals were obtained.

### 2.1. Calibration and detection limit

Once the optimal experimental conditions were found for the analysis of arsenic by means of Ach/SPCE, a calibration was performed using a least-median-squares regression (LMS) to detect the existence of anomalous points [21], which might have led to incorrect adjustments altering the sensitivity and the detection limit. Several calibration curves were obtained in the concentration range 1 × 10^−8^ to 1 × 10^−7^ M for AcH/SPCE.

A key feature of any analytical method is its detection limit; the smallest concentration of the analyte that can be detected to a specified degree of certainty. The detection limit, based on the variability of eight samples with a 1 × 10^−8^ M concentration of As(III), was evaluated according to [22] and ISO 11843-2 [23]. At the chosen probability level of 5% (*α* = *β* = 0.05), the detection limit was 1.1 × 10^−8^ M.

### 2.2. Precision

The precision of the developed method was calculated in terms of repeatability and reproducibility. In order to calculate the repeatability of the method, successive amperometric measurements with the same electrode surface, conditioned at 4 °C for 1 h in a Britton-Robinson buffer solution pH 7 between experiments, were tested. Sets of three successive calibrations for arsenic were realized yielding a relative standard deviation for their slopes of 3.4%. Likewise, the reproducibility of the amperometric signal was checked using the slopes of three regressions carried out with different electrode surfaces. The RSD values obtained were 4.0%. These results suggest that the fabrication procedure of the Ach based biosensors is reliable, and allows reproducible electroanalytical responses to be obtained with different electrodes constructed in the same way.

### 2.3. Interferences

**Figure 2.**
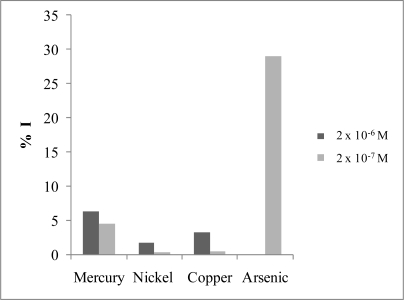
Percentage of inhibition % I caused by different metals. E_ap_ = 0.6 V *vs*. Ag/AgCl, Britton-Robinson buffer pH = 7 and acetylthiocholine iodide concentration 3.64 × 10^−4^ M.

The action of As(III) as an Ach inhibitor is not specific. A number of possible interfering metals ions (Zn(II), Cu(II), Cd(II), Ni(II), Fe(III), Pb(II), Hg(II), Cr(VI) and Cr(III)) were investigated. Ni(II) and Cu(II) at concentrations higher than 2 × 10^−6^ M were found to have some influence, causing a fall in the acetylthiocholine iodide response. But, the most important interference was caused by Hg(II), which is detectable at mercury concentrations higher than 2 × 10^−7^ M. This interference study was carried out in absence of As(III). The described inhibition effect can be seen in Figure 2. The figure represents the percentage of inhibition caused by two different concentration levels of the interfering ion. The degree of inhibition % I was calculated on the basis of the relation: % I = ΔI × 100/I_o_, where ΔI is the difference between the value of substrate registered amperometric signal in absence, I_o_, and in presence of interference. In this Figure, the high inhibition caused by a low concentration of arsenic is also represented.

### 2.4. Analytical application

*Determination of As(III) in spiked tap water samples.* The developed disposable biosensor was used for the analysis of As(III) in spiked tap water samples by standard addition (concentration of As(III) 1.00 μM). The concentration found in the tap water sample was 1.04 ± 0.05 μM (*n* = 6, *α* = 0.05, RSD = 4.1%). This value closely agrees with the real one.

*Determination of As(V) in a certified water sample.* The proposed method was also successfully applied to the determination of arsenic(V) in a certified water sample by standard addition. Since As(V) is not an Ach inhibitor, a previous reduction stage of this specie is necessary in order to determine the concentration of this sample by the developed method. The reduction process was carried out by mixing 100 μL of the certificate sample (1.336 × 10^−2^ M) with 100 μL of 0.1 M sodium thiosulfate and water until a final volume of 10 mL. The mixture was left to react for 70 minutes at room temperature. The arsenic concentration obtained in the certified water sample was (1.361 ± 0.095) × 10^−2^ M (n = 6, α = 0.05, RSD = 6.7%). This result is in good agreement with that supported by manufacture (1.336 ± 0.006) × 10^−2^ M.

## 3. Experimental Section

### 3.1. Chemical reagents

Ach (E. C. 3.1.1.7, 1047 U/mg from electric eel) purchased from Sigma (Steinheim, Germany), acetylthiocholine iodide and N-cyclo-hexyl-N’-[2-(N-methylmorpholino) ethyl]carbodiimid 4 toluensulfonate (Fluka, Buchs, Switzerland) were used. Sodium thiosulfate (Na_2_S_2_O_3_·5H_2_O) was obtained from Merck (Darmstadt, Germany). Potassium chloride was purchased from Panreac (Barcelona, Spain). Sodium (meta)arsenite (NaAsO_2_, 99%) was purchased from Fluka (Buchs, Switzerland). Arsenic acid (H_3_AsO_4_, 1,002 ± 5 mg L^−1^) solution CertiPur® was obtained by Merck (Darmstadt, Germany). Britton-Robinson buffer with different pH values was used. All the reagents were used without further purification. All solutions and subsequent dilutions were prepared using deionized water obtained with a Barnstead NANO Pure II system.

As(III) stock solutions (10 mM) was prepared fresh daily from NaAsO_2_ (0.013 g NaAsO_2_ dissolved in 10 mL deionized water).

The electrochemical system was produced using polymeric commercial inks. Electrodag PF-407 A (carbon ink), Electrodag 6037 SS (silver/silver chloride ink) and Electrodag 452 SS (insulator ink) were obtained from Acheson Colloiden (Scheemda, Netherlands).

### 3.2. Instrumentation

Hand-made screen-printed electrodes were produced with a DEK 248 printing machine (DEK, Weymouth, UK) using polyester screens with appropriate stencil designs mounted at 45° to the printer stroke.

Electrochemical measurements were recorded using a μAutolab type III electrochemical system with GPES software (EcoChemie, Utrecht, Netherlands).

The pH of the solutions was measured with a Crison Model 2002 (Barcelona, Spain) pH meter.

### 3.3. Software

Data analysis was performed using a STATGRAPHICS PLUS software package [24], and PROGRESS [21] for the robust regression.

### 3.4. Construction of the biosensors

**Figure 3.**
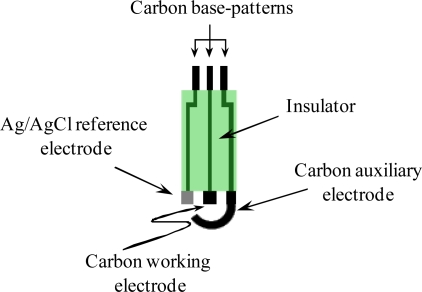
Diagram of the three-electrodes screen-printed configuration used in the fabrication of the biosensors.

**SPECs preparation**. Hand-made screen-printed electrodes (working electrode area, 4 mm^2^) were used in the electrochemical determination of arsenic. For the construction of the screen-printed electrodes (Figure 3) successive layers of different inks were printed onto a polyester film substrate using three different screens with appropriate stencils to transfer the required design following the printing procedure described in previous works [25,26].

**Electrode cleaning**. Before utilization, the SPCEs, working and counter electrodes were softly polished during almost one second with a SiC-paper No 4000 disc (Struers, Copenhagen, Denmark). After polishing, the electrode system was washed with water. Then, the working electrode surface was activated by recording 40 cycle voltammograms between 2 and −2 V, scan rate, 100 mV s^−1^, in a 0.1 M KCl solution.

**Acetylcholinesterase immobilization in SPCEs**. Ach was immobilized by covalent linkage on the working electrode surface. The mode of preparation of the Ach amperometric sensor was adapted from previously published reports [18,19]. 5 μL of a 0.05 M N-cyclo-hexyl-N’-[2-(N-methylmorpholino) ethyl] carbodiimid 4 toluensulfonate solution prepared in Britton-Robinson pH 7 were deposited on the working electrode surface. After 80 min activation at room temperature, 5 μL of Britton-Robinson buffer (pH 7) solution containing 2.5 mg/mL of Ach were dropped on the working electrode surface. During this activation step [27] the reaction between carboxilic groups and carbodiimid gives rise to a more active substrate for its reaction with the amine groups of the enzyme. Then, the electrode was kept at 30 °C for 2 h. Finally, the electrode surface was rinsed with buffer solution.

**Arsenic determination procedure**. The Ach biosensors were placed in the electrochemical cell containing 5 mL of Britton-Robinson buffer solution. An adequate potential was applied, and once a steady-state current was set, a defined amount of acetylthiocholine iodide stock solution was added to the measuring cell. A large oxidation current was observed, and a plateau corresponding to the steady-state response was reached. Then, fixed portions of the arsenic stock solution were added consecutively, being reached each time a plateau. The addition of arsenic solution resulted in a current decrease of the oxidation signal of acetylthiocholine iodide proportional to the amount of arsenic added.

Between its calibration setting the biosensor was conditioned by dipping in a Britton-Robinson buffer (pH 7) solution at 4 °C for 1 h.

**Biosensor storage**. Enzyme electrodes were storage in a Britton-Robinson buffer (pH 7) solution at 4° C. Under these conditions, the biosensor was stable for arsenic determination for at least 15 days.

## 4. Conclusions

The developed Ach/SPCE biosensor allows the selective and sensitive amperometric determination of As(III). The proposed method shows high reproducibility and repeatability in the determination of this metal in water samples.

Comparing this study with previous described works, the proposed method offers several advantages. Firstly, the disposable character of the SPCE should be highlighted. Also, the construction procedure used in this work is simpler and it minimizes considerably the amount of enzyme and other reagents used in the immobilization step. On other hand, the potential used for arsenic determination, +0.6 V, is lower than the potential used in previous reported works. This fact could improve the selectivity of the proposed method.

Finally, this method is also potentially useful for redox speciation analysis of arsenic.
